# Classification of Pigs with Tail Lesions from Different Farrowing and Rearing Systems during Rearing and Fattening Period

**DOI:** 10.3390/ani9110949

**Published:** 2019-11-11

**Authors:** Maria Gentz, Anita Lange, Sebastian Zeidler, Imke Traulsen

**Affiliations:** 1 Department of Animal Sciences, Livestock Systems, Georg-August-University, Albrecht-Thaer-Weg 3, 37075 Göttingen, Germany; anita.lange@agr.uni-goettingen.de (A.L.); imke.traulsen@uni-goettingen.de (I.T.); 2 Department of Animal Sciences, Breeding Informatics, Georg-August-University, Margarethe-von-Wrangell-Weg 7, 37075 Göttingen, Germany; sebastian.zeidler@uni-goettingen.de

**Keywords:** classification, tail lesions, pigs, rearing system, farrowing system, docked tails, undocked tails, stress, tail biting, cluster analysis

## Abstract

**Simple Summary:**

Tail biting is a well-known problem in pig production, whereby animals with tail lesions suffer from pain, infections, and reduced feed intake. Controlling tail biting could have a positive effect on animal welfare and on the economic viability of farms. A classification of typical traits of pigs with tail lesions could provide the opportunity to protect the pigs. A combined parameter based on frequency and duration of tail lesions was created to find out whether biologically relevant groups can be separated by cluster analysis. The results show that the created lesion parameter was suitable to describe the degree of impairment of the pigs. However, not all pigs were affected to the same extent by tail biting. The high impact of the docking status and the reduction of tail lesions by more space allowance was shown.

**Abstract:**

The aim of the present study was to classify and characterise pigs with tail lesions using a combined parameter based on the frequency and duration of tail lesions and to find out whether biologically relevant groups could be separated by cluster analysis. Pigs (n = 677, 50% docked, 50% undocked) from three farrowing systems, as follows: (1) Conventional farrowing crate (FC), (2) free farrowing (FF), and (3) a group housing lactating sows (GH), were divided into two rearing systems as follows: (1) A conventional system (CONV) and (2) a wean-to-finish (W-F) system. Within 18 assessment weeks, starting after weaning, animal tail lesions were recorded individually. The animals were characterised into five lesion groups, as follows: (I) No lesions to (V) many long lasting lesions. The separability of the predefined lesion groups was checked by an animal individual lesion parameter. By using a k-means cluster analysis, it was shown that the docking status was the mainly affected parameter on the tail lesions. The separation of the groups only succeeded for the most distinct groups, I and V. The high impact of the docking status and the reduction of tail lesions by more space allowance was shown. More characterising information for the individual pigs would improve the separability of the lesion groups.

## 1. Introduction

Tail biting is a huge and well-known problem in pig production. A lesion can range from mild bite marks, with or without punctuation of the skin, up to a complete tail loss [1]. If a tail lesion occurs, it results in pigs suffering from pain, and more symptoms, e.g., reduced weight gain and a higher risk of infection diseases, which results in a need for medical treatment [2,3]. Tail lesions can also lead to economic losses at slaughter, carcass condemnation, and secondary infections [4]. Pigs show individual reactions to different stressors such as climatic and light conditions [5], sex [6], husbandry environment [7], feeding [8], and group size [9], among others. Tail biting does not necessarily lead to lesions. Taylor et al. (2010) [10] described three different types of tail biting behaviour, whereby the first type describes a gentle and harmless chewing on the tail of another pig, where the tail remains complete and shows no lesions [3], but can also result in tail lesions which can be responsible for a tail biting outbreak [10,11]. The second type of tail biting is energetic tail biting, which can be triggered by deficiencies in the abundance or accessibility of water, feeding, or space. The third type is a compulsive and abnormal behaviour of individual animals whose cause is still unknown [10]. 

In order to work out profiles within groups, a cluster analysis is often carried out [12,13]. For example, in human fields, this method is used in gene expression analysis [12], thermography [13], or for motivation profiles of athletes [14]. The k-means cluster algorithm is used to find optimal distribution profiles or to work out specific character traits [12,13]. In addition, the relationship between single groups can be ranged [14]. A k-means cluster analysis is preferred, as this method is particularly suitable for large amounts of data and the number of clusters generated can be predefined. After cluster analysis, the identified relevant groups can be analysed in detail by several tests to separate and describe these groups.

Different types of tail biting can lead to different types of wounds [10]. There are small wounds that heal up quickly and large wounds that require more time [10,15]. Consequently, the degree and duration of tail lesions differ more or less. This means that pigs can be divided into animals without lesions, with few, and with many tail lesions. In addition, the intensity of the wound determines the duration of healing. 

A reduction of stressful factors and an optimisation of the housing conditions can increase animal welfare and help to reduce tail biting as a coping strategy [16]. For example, early socialisation was shown to help piglets to handle stressful situations better [17]. Farrowing and rearing systems with a higher space allowance, and without any fixation of the sow, may improve the socialisation level of piglets and the welfare of sows [18].

The aim of this study was to classify and characterise pigs using a combined parameter, based on their frequency and duration of tail lesions, and to find out whether biologically relevant groups could be separated by cluster analysis. As influencing factors, docking status, rearing system, farrowing system, and weights at weaning as well as at the end of rearing were considered.

## 2. Materials and Methods

### 2.1. Animals and Housing

**Figure 1:**
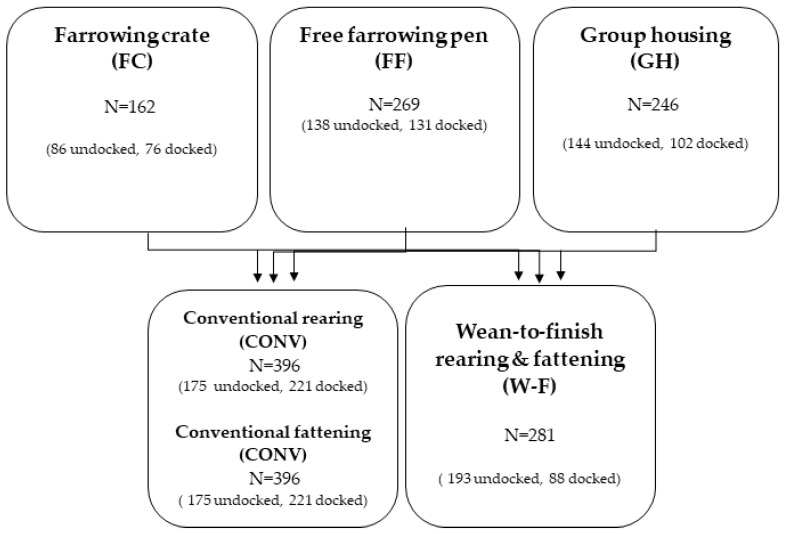
Schematic overview of the 3 × 2 × 2 factorial experimental design.

The dataset comprised measurements of 1252 pigs [18], where 677 animals provided complete measurements in 18 subsequent weeks. The data was accquired at the research farm Futterkamp at the chamber of Agriculture of Schleswig-Holstein, located in northern Germany. Within seven batches, 309 pigs were housed with docked tails (45.64%) and 368 pigs were left undocked (54.36%). Males were raised as intact males. For farrowing, sows were allocated to three farrowing systems, as follows: A conventional farrowing crate (FC), a free farrowing system (FF), and a group housing system of lactating sows (GH) (Figure 1). In the FC and FF systems, the sows were single housed. In the FC, sows were fixed permanently, and in the FF system, sows could move freely and interact with their piglets, except for one batch, in which the sows were fixed for three days post-partum to minimise the crushing of the piglets (piglets treated as being FF). In the GH system, ten sows were housed together. Until five days post-partum, the sows were single housed in a free-farrowing pen. Afterwards, the sows and their piglets were commingled, joining a running area and their pens.

Figure 1 shows an overview of the 3 × 2 × 2 factorial experimental design. After 27 days post-partum, the piglets were weaned and divided randomly within the farrowing system into two different rearing systems, as follows: A conventional rearing system (CONV_rearing_, 0.44 m^2^/pig) and a wean-to-finish system (W-F, 0.89 m^2^/pig). The W-F pigs were grouped by sex and the CONV_rearing_ pigs were grouped mixed-sex. Pigs of the CONV_rearing_ group were regrouped and rehoused for finishing and sorted by sex after 40 days of rearing (CONV_fattening_), while the W-F pigs were raised as intact groups until the end of the study (week 18 after weaning). The CONV_fattening_ pigs had 0.89 m^2^ per pig and their pens were identical in structure to those of the W-F pens. In CONV_rearing_, the piglets were housed in groups of 13 pigs and in W-F, the piglets were housed in groups of 14 pigs. The slat width on all floors (13 mm, tread area = 67 mm) was determined in a special permit (V242-226720/2015 (8-1/16)) to allow the pigs to be kept in the same pens from weaning to the end of fattening. The pigs were fed ad libitum with conventional dry feed adjusted to their age. During their whole life, the pigs always had access to at least one or several different enrichment materials; for example, pieces of wood, ropes, jute bags, or troughs filled with dried peas and grass pellets. When an outbreak of tail biting occurred, pigs were offered additional or renewed enrichment material. Additionally, victims of tail biting that were severely injured were excluded from the study (N = 19). Evident offenders were also rehoused and excluded (N = 9). Pigs with medical treatable lesions stayed in their pens and were scored consecutively. More details on housing systems can be found in Gentz et al. (submitted) [19].

All of the animals were housed in accordance with EU (European Directive 2008/120/EC) and national law (Animal Welfare Act (18/05/2016), Animal Welfare and Animal Husbandry Ordinance (05/02/2014)).

### 2.2. Data Collection

The weaning weight and the 40-days-weight were collected individually. The two weights were included in the evaluation. 

Starting one day after weaning, the piglets’ tails were scored individually once a week until assessment at week 18 using a modified Schwarzenauer key [20]. From the collected data, the tail lesions were used for the present study. A tail “lesion” means that the tail has slight scratches or bite marks, a “severe lesion” shows deeper flat lesions, and a “very severe lesion” would be a deep flat lesion which is greater than 2 cm.

For the statistical analysis, the tail lesion score was summarised due to the low occurrence of severe lesions into “no lesions” (0) and “lesions” (1). Each pig was scored for each assessment week, whether a tail lesion was observed or not. 

### 2.3. Statistical Analysis

**Figure 2:**
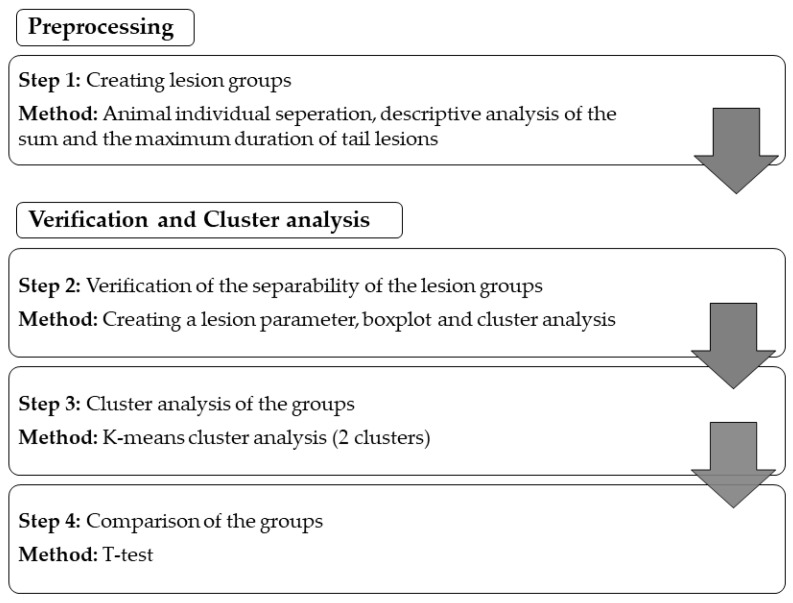
Workflow of the statistical analysis.

The statistical language R [21] was used to analyse the collected data. The statistical analysis was conducted in different steps, which are explained in the following scheme (Figure 2) (oriented at the method of Lukashin and Fuchs, 2001 [12]). 

#### 2.3.1. Preprocessing 

Step 1: Creating Lesion Groups

The aim of this step was to create a single animal individual lesion parameter. First, an analysis of the frequency of score “1” and the maximum duration of consecutive score “1” was carried out (Table 1). The frequency of score “1”, which represents the sum of score “1” over all 18 assessment weeks, was calculated. In addition, the maximal duration of consecutive score “1” was calculated. This was used as an indicator of the impact of tail lesions. Table 1 shows an example for the scoring of five different pigs.

Pig 1 had no score “1” over all the assessment weeks, which means that the frequency of score “1” was zero and the maximum duration as well. For pig 2, three single scores of “1” were observed, which led to a frequency of score “1” of three and a maximum duration of one, because there was no more score “1” in a row. Pig 3 had a score of “1” during weeks six, seven, and twelve. This means that the frequency of score “1” was three and the maximum duration, as there were two successive score “1” during weeks six and seven, would be two. Pig 4 had three successive and one single score “1” (frequency = 4; maximum duration = 3). Pig 5 had a frequency of score “1” of 14 and a maximum duration of ten. This means that not all 14 scores of “1” were in successive weeks, but ten were successive and four were at any other time. 

**Figure 3:**
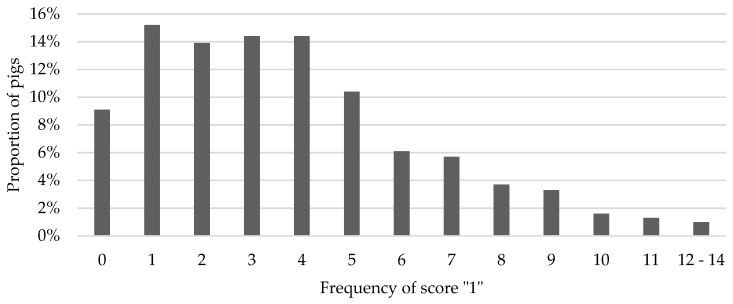
Distribution of the frequency of score “1” of tail lesions per pig over rearing and finishing periods.

Figure 3 shows the distribution of the frequency of score “1”. A total of 77.4% of the pigs had five or less scores of “1” and 22.6% suffered from more scores of “1”. Less than ten percent of the pigs did not have any score “1” at all. The percentage of pigs with ten or more scores of “1” was 3.9%.

In order to separate these pigs, the maximum duration score was used to determine how many consecutive weeks a pig had score “1”. 

**Figure 4:**
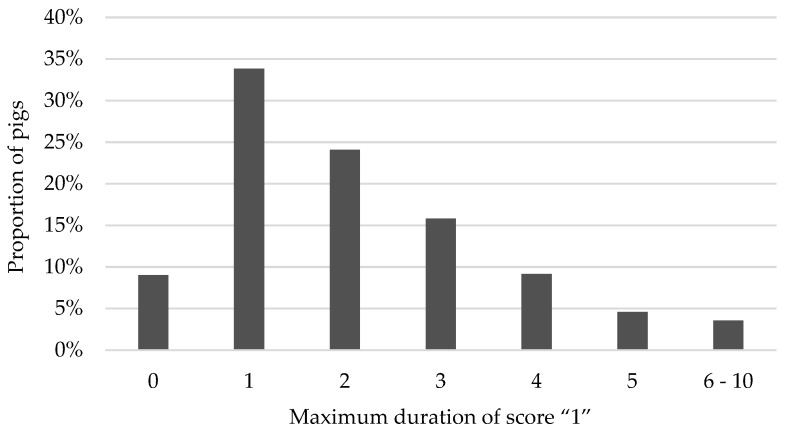
Distribution of the maximum duration of score “1” per pig over rearing and finishing periods.

Figure 4 shows the distribution of the maximum duration of a tail lesion score “1”. Scores of “1” that lasted longer than six and for a maximum of ten weeks were summarised due to rareness. 

Based on the frequency of score “1” and the maximum duration, five lesion groups were formed (Table 2). Each pig could only be found in one group. The primary criterion for the group was the frequency of score “1” and the maximum duration worked as second criterion. The duration was divided into short and long, whereby short meant 1/3 of the maximum duration within their lesion group and long meant 2/3 corresponding to the maximum duration within their lesion group. Therefore, the pigs with tail lesions could be divided into the following groups: (I) No lesions, (II) few short lesions, (III) few long-lasting lesions, (IV) many short lesions, and (V) many long-lasting lesions. 

The first group consisted of pigs without tail lesions, meaning no scores of “1” over all the 18 assessment weeks (I: 61 pigs, 9.01%). In the second and third group, there were pigs with few tail lesions (frequency of score “1”: 1–3) (II: 204 pigs, 30.13%; III: 90 pigs, 13.29%) and the fourth and fifth group consists of pigs with many tail lesions (frequency of score “1”: 4–14) (IV: 205 pigs, 30.28%; V: 117 pigs, 17.28%). 

#### 2.3.2. Cluster Analysis

Step 2: Verification of the Separability of the Lesion Groups

In Step 2, the separability of the predefined lesion groups was checked. To classify biologically meaningful groups associated with the used docking status, the rearing and the farrowing systems, the lesion groups had to be mostly non-overlapping. In order to test the separability of the individual lesion groups, an animal individual lesion parameter (λ) was calculated.

$$\lambda =\{\begin{array}{c} 0,\;\mathrm{if}\;\mathrm{frequency}\;\mathrm{of}\;\mathrm{score}\;“1”=0\;\wedge \mathrm{maximum}\;\mathrm{duration}=0\\ \frac{\mathrm{frequency}\;\mathrm{of}\;\mathrm{score}\;“1”\;+\;\mathrm{maximum}\;\mathrm{duration}\;}{\frac{\mathrm{frequency}\;\mathrm{of}\;\mathrm{score}\;“1”}{\mathrm{maximum}\;\mathrm{duration}}} \end{array}$$

**Figure 5:**
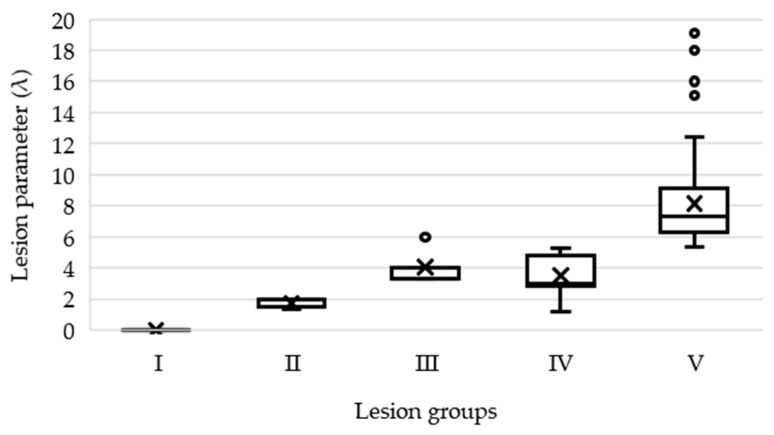
Boxplot analysis of the lesion parameter (λ) for each lesion group.

Figure 5 shows a boxplot of the lesion parameter for the different lesion groups.

**Figure 6:**
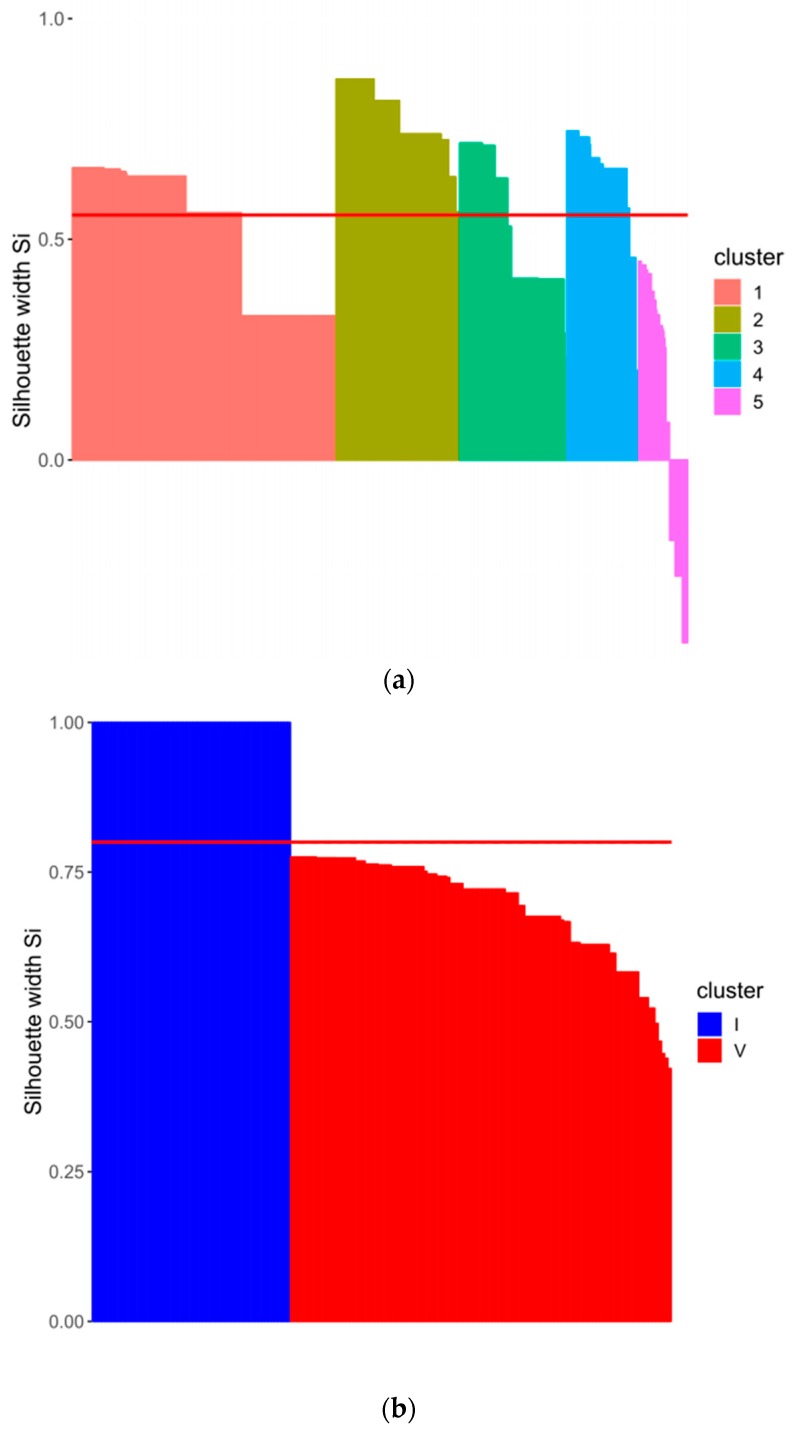
(**a**) Silhouette plot for five k-means cluster groups to show the non-separability. The red line shows the average quality of the clusters. (**b**) Silhouette plot for two k-means cluster groups to show the separability. The red line shows the average quality of the clusters.

The boxplots show that groups I and V were completely separable from each other. Groups II, III, and IV were difficult to distinguish due to their overlapping lesions scores. In addition, the k-means-clustering algorithm (k = 5, 1000 iterations) was applied on the data to check for the plausibility of the separation and to validate the lesion groups. The silhouette plot (Figure 6a) underlines the difficulties in separating the five groups, whereas cluster groups 2–4 show low average silhouette scores and cluster group 5 shows negative silhouette scores. To improve the degree of separation, the clusters were modified (increased and decreased), which showed an improved separability in the case of two clusters only. The subsequent analysis was focused on groups I and V, which represented no lesion animals and animals with many long-lasting scores of “1” (Figure 6b).

Step 3: Cluster Analysis of Most Distinct Groups

**Figure A1:**
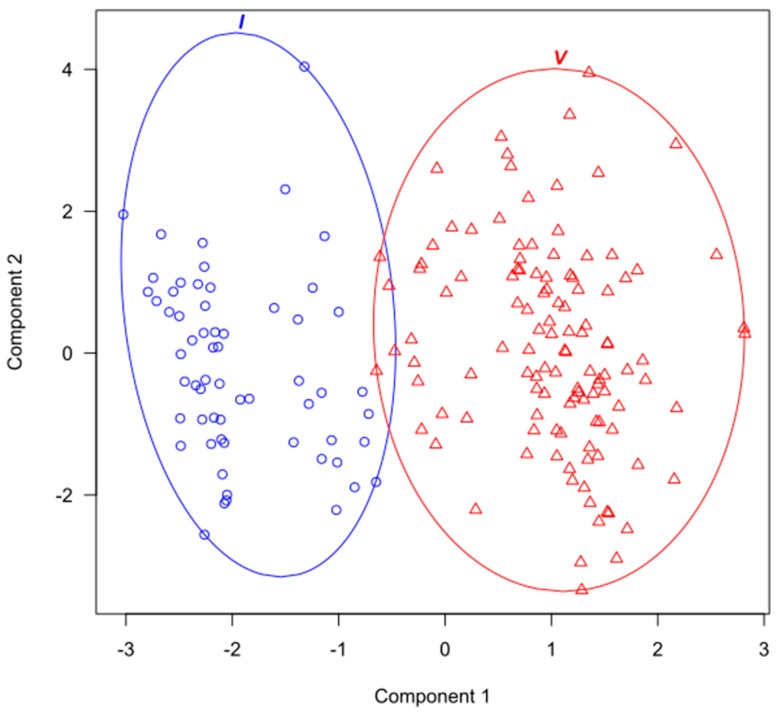
Cluster plot of the two separated groups I (blue) and V (red). The cluster analysis resulted in two nearly non-overlapping groups. The pigs without lesions (group I) were clearly separable from the pigs with many long-lasting scores of “1” (group V).

With help of λ, a k-means cluster analysis [21,22] for groups I and V was conducted. This iterative method of minimizing the within-class sum of squares for a given number of clusters was used to test the classification into the specific lesion groups. The visualization was conducted using the cluster package [23]. Figure 6b shows, that the data can be separated in two distinct groups, which represent the two lesion groups I and V (see Appendix A Figure A1). 

Step 4: Comparison of the Non-Overlapping Groups

In the last step, the non-overlapping groups I and V were compared. The docking status, the rearing system, and the farrowing systems were analysed according to the relative frequency of pigs. In order to investigate the significant differences of the weights at weaning and after rearing a t-test was applied. 

## 3. Results

The distribution of pigs within the lesion groups (I and V) per docking status, rearing system, and farrowing system is shown in Table 3. Group I consisted of twice as many docked than undocked pigs, whereas group V shows a reversed distribution. The Χ^2^ test showed that groups I and V, as well as the docked and undocked pigs, were significantly different (*p* < 0.05). In general, the distribution of the pigs within group V of the rearing and farrowing systems of group V were very similar (Rearing system: CONV = 16.67, W-F = 18.15%; farrowing system: FC = 15.43, GH = 17.07, FF = 18.59%). There were more W-F pigs than CONV pigs in each group. The proportion of FC and GH pigs in group I was lower than that in group V. Among the FF pigs, group V contained about one-third more animals than group I.

The comparison of the different weight groups only resulted in significant differences in the group of undocked-CONV-FF animals with regard to the average weaning weights (I = 6.63 kg, V = 8.35 kg) and the average weight at the end of rearing (I = 22.00 kg, V = 26.40 kg), where the pigs of lesion group V were heavier. No other animals comparing group I and V differed significantly. The average weaning weight was 7.96 kg (min = 6.35 kg, max = 9.35 kg) and the average difference between groups I and V was 0.93 kg. Regarding the weight at the end of rearing, the average weight was 25.18 kg (min = 21.20 kg, max = 28.50 kg), whereby the groups I and V differed by 1.33 kg on average.

## 4. Discussion

The lesion parameter λ was used as a continuous and non-prioritising parameter to define the intensity of tail lesions. It combined the frequency and the duration of tail lesions on an equal level. This parameter might be a useful help to analyse tail lesions in further studies.

A cluster analysis is often used for profiling [12,13,14], as was also done in the present study. The results show that a reliable clustering could only be performed in groups I and V, where the differences of the frequency and the durations were the highest. Groups II, III, and IV were too similar to work out specific traits. In order to figure out the impact of the individual animals and their tail lesions, other traits may have to be considered. 

The predefinition of the number of clusters gave the possibility to investigate a specific biologically grounded hypothesis [24]. In an experimental design without expectation of a number of clusters, this analysis requires a longer preliminary work. This includes first hierarchical clustering followed by non-hierarchical clustering [14]. In this study, the k-means clustering made it possible to separate very similar groups respectively to emphasise the separable groups. 

Although a total of 1252 fattening pigs were investigated in this study, it was only possible to examine 677 pigs after strict filtering. To assure a representative animal number in both groups (I, V), an enormous number of assessed animals or simulating techniques might be an option. In addition, it should be noted that the numbers of pigs were not balanced concerning the distribution among the husbandry systems, as well as the docking status, and this leads to limitations in the interpretation of the results. The unbalanced data set influenced the comparison of the lesion groups. The very dominant effect of the docking status covered the other effects. An investigation of only undocked pigs might have resulted in other significances, but the low number of animals would have led to other methodical problems. This method could be improved by using more animal individual characterising traits, such as behaviour or genetics, to make them unique. Since various factors affect the pigs and tail biting is a multifactorial problem, it is difficult to determine the most influential traits to avoid tail lesions [3].

Docking is one of the most commonly used methods to reduce tail biting [3]. It reduces the risk for tail biting related lesions but can neither prevent it completely [25] nor eliminate the causes for the abnormal behaviour. The literature still does not provide clear reasons why docked pigs get less tail lesions [1], but one hypothesis is nerve regeneration. Docking creates a higher sensitivity at tail ends and this benefits in a quicker reaction [26,27]. In addition, the attractiveness of a docked tail is lower [28]. The significant effect of tail docking might cover the effects of the rearing and the farrowing system. Nevertheless, differences (not significant) within the husbandry systems show, which might be the better way of housing.

Findings for W-F pigs were in line with the literature. Pigs which were not regrouped and not rehoused had fewer tail lesions and might suffer from less stress, resulting in fewer tail lesions [29]. The present study can support the results of Beattie et al. (1996) about increasing space allowance, which resulted in a reduction of tail biting behaviour among growing pigs [3,30]. The advantages of higher space allowance and loose housing can also be seen from the FF pigs. The frequency of FF pigs in group I was particularly high, which led to the conclusion that the farrowing system has a positive effect on how piglets dealt with their post-weaning environment [18]. Better socialisation of the piglets also seemed to result in a reduction of aggression in their future life [17].

With regard to tail biting, future studies should include more information about the age of the wound to distinguish fresh from old healing or reopened lesions to gather information about the healing process. This would offer the possibility for further improvement in the analysis, especially to analyse whether a pig gets new lesions every week or if there are problems with wound healing. 

## 5. Conclusions

This study shows that not all pigs were affected to the same extent by tail biting, which can generally be measured by the lesion parameter. Groups of pigs without tail lesions could be separated from pigs with lesions. The cluster analysis can help to analyse and separate the lesion groups. To enhance the analysis, a larger dataset with only undocked pigs is needed. The availability of more individual animals and unique traits might improve the applicability of the present analysis procedure. Although several traits of the pigs with tail lesions were assessed, it was only possible to name significant differences regarding docking status to characterise the groups.

## Figures and Tables

**Table 1:** Examples of the tail lesion scoring, frequency of score “1”, and maximum duration (1 = a tail lesion was scored; 0 = no tail lesion was scored). ^1^ Frequency of score “1”; ^2^ maximum duration of score “1”.

	Assessment Weeks																	Freq. ^1^		Max. Dur. ^2^
	1	2	3	4	5	6	7	8	9	10	11	12	13	14	15	16	17	18		
**Pig 1**	0	0	0	0	0	0	0	0	0	0	0	0	0	0	0	0	0	0	**0**	**0**
**Pig 2**	0	0	0	1	0	0	0	1	0	0	0	1	0	0	0	0	0	0	**3**	**1**
**Pig 3**	0	0	0	0	0	1	1	0	0	0	0	1	0	0	0	0	0	0	**3**	**2**
**Pig 4**	0	0	0	0	0	0	0	1	1	1	0	0	0	0	1	0	0	0	**4**	**3**
**Pig 5**	1	1	1	1	1	1	1	1	1	1	0	0	1	1	0	1	0	1	**14**	**10**

**Table 2:** Lesion groups (LG) built of the frequency of score “1” and the maximum duration of score “1”. Note that each pig was in one group only.

Groups	Freqency of Score “1”	Max. Duration of Score “1”	N ^1^
**I** No lesions	0	0 weeks	61 pigs
**II** Few lesions of short duration	1–3	1 week	204 pigs
**III** Few lesions of long duration		2–3 weeks	90 pigs
**IV** Many lesions of short duration	4–14	1–3 weeks	205 pigs
**V** Many lesions of long duration		4–10 weeks	117 pigs

^1^ 18 assessment weeks for each pig; Exemplary pigs: See Table 1 (LG I Pig 1, LG II Pig 2, LG III Pig 3, LG IV Pig 4, and LG V Pig 5).

**Table 3:** Distribution of the characteristics of the pigs within the lesion groups I and V, related to all pigs of the specific group (Figure 1). Note that the significant differences were calculated with absolute numbers of animals.

		Lesion Group		
		I (n = 61)	II–IV (n = 499)	V (n = 117)
**Docking status**	undocked	5.43% ^a^	83.16%	11.41% ^b^
	docked	13.27% ^c^	80.26%	6.47% ^d^
**Rearing system**	CONV	6.57%	76.76%	16.67%
	W-F	12.46%	69.39%	18.15%
**Farrowing system**	FC	6.79%	77.78%	15.43%
	FF	11.90%	69.51%	18.59%
	GH	7.32%	75.61%	17.07%

^a–d^ Different letters indicate significant differences of the groups (*p* < 0.05).
